# Effect of Alexandrite Laser Hair Removal on the Activity of Systemic Lupus Erythematosus

**DOI:** 10.31138/mjr.160724.eoa

**Published:** 2024-10-22

**Authors:** Elias Salimi, Shirin Assar, Mahsa Rashidi, Dena Mohamadzadeh

**Affiliations:** 1 Clinical Research Development Centre, Imam Reza Hospital, Kermanshah University of Medical Sciences, Kermanshah, Iran; 2 Student Research Committee, Kermanshah University of Medical Sciences, Kermanshah, Iran

**Keywords:** SLE, hair removal, Alexandrite laser

## Abstract

**Objective:**

While ultraviolet light is a well-known environmental trigger of Systemic lupus erythematosus (SLE), it is unknown whether other spectra of light including infrared could affect SLE activity. This study aimed to evaluate the effect of laser hair removal which emits red and infrared light on the activity of SLE.

**Method:**

20 patients with SLE were enrolled. Six monthly sessions of laser hair removal with Alexandrite laser were done. Demographic and clinical data were recorded. SLE disease activity index (SLEDAI-2K), serum levels of Anti-ds-DNA, C3, C4, and CH50 complement levels, and white blood cell and platelet counts were measured before and after the laser course to investigate the activity of SLE.

**Results:**

Most of the participants were female (90%) with a mean age of 32.65. Prednisolone was the most commonly used medication (95%) followed by hydroxychloroquine (90%). The most common skin types according to Fitzpatrick’s classification were types II and III. We found no significant differences between the SLEDAI-2K score, and serum level of Anti-ds-DNA, C4, and C3 before and after the laser hair removal.

**Conclusion:**

Laser hair removal is safe and does not affect the activity of SLE and might not induce disease exacerbation.

## INTRODUCTION

Systemic lupus erythematosus (SLE) is a systemic chronic inflammatory autoimmune disease that can involve almost any organ including skin, joints, kidneys, liver, lungs, and the nervous system. Clinical and immunological manifestations are heterogeneous among different individuals. The periods of remissions and exacerbations are characteristic of SLE. Both genetic predisposition and environmental triggers play a role in disease development and flare-ups. The environmental triggers include infectious agents, cigarette smoking, exogenous sex hormones, certain medications, chemicals, and ultraviolet light. Oxidative stress induced by the mentioned triggers reduces DNA methylation in CD4^+^ T-cells which promotes autoimmunity.^1,2^ It is known that ultraviolet B induces apoptosis of dermal cells such as keratinocytes leading to the release of a large amount of autoantigens and pro-inflammatory cytokines. In addition, it may alter DNA and intracellular proteins to make them antigenic.^1–3^

Hair growth in unwanted places has become a typical cosmetic concern and laser hair removal has been increasingly used in recent years. Available laser devices emit a red and infrared spectrum of light which is absorbed by melanin and destroys hair follicles. Alexandrite laser emits a 755 nm wavelength that is in the range of infrared light and is more suitable for Fitzpatrick skin types II to III. Damage to melanocytes and other dermal cells has been shown previously,^4^ which raises the concern of the possibility of SLE increased activity after laser hair removal. It is unknown whether the red and infrared spectrum of light could affect DNA and intracellular proteins. As far as we know, no previous study was done to answer this question. The aim of the present study was to evaluate the effect of the Alexandrite laser on the activity of SLE.

## METHOD

This study was conducted in 2022 in Kermanshah, Iran. We chose 20 patients with systemic lupus erythematosus (SLE) from those attending the rheumatology clinic of the Kermanshah University of Medical Sciences (KUMS). The inclusion criteria were as follows: 1) Satisfying the 2019 American College of Rheumatology classification or European League Against Rheumatism (ACR/EULAR) criteria for SLE^5^; 2) having the desire to undergo hair removal laser for unwanted hair. The exclusion criteria were 1) previous history of severe histamine reactions or allergy to lidocaine, 2) having bleeding disorders, 3) the presence of sunburn or recent surgery on the skin, and 4) a tendency of keloid formation, 5) high disease activity level based on the SLEDAI-2K score. The local Clinical Research Ethics Committee approved the study protocol (approval number IR.KUMS.MED.REC.1401.034).

The following demographical and clinical data were recorded for each patient before the laser procedure: age, sex, education level, marital status, underlying diseases except SLE, medications for SLE, and skin phenotype of the patients according to the Fitzpatrick classification^6^ (the skin phenotype’s definition was as follows: type one: pale white skin which always burns and does not tan, type two: fair akin which burns easily and tans poorly, type three: darker white skin which tans after the initial burn, type four: light brown skin which burns minimally and tans easily, type five: brown skin which rarely burns and rans darkly easily, type six: dark brown or black skin which never burns but always tans dark), skin thickness, systemic lupus erythematosus disease activity index (SLEDAI-2K), serum levels of Anti-ds-DNA, C3, C4 and CH50 complement levels, white blood cell, and platelet counts.

The patients underwent six monthly sessions of whole-body laser hair removal with Alexandrite Apogee laser using an average fluence of 10 to 20 J/cm^2^, an 18 mm spot size at either a 20 to 40 ms pulse duration, and 2 ms pulse width. At the end of the laser sessions, SLEDAI-2K was calculated and laboratory investigations (Anti-ds-DNA, C3, C4, and CH50 complement levels, white blood cell, and platelet counts) were performed again. The results of the mentioned variables before and after the laser procedure were compared to evaluate the possibility of an increase in the activity of SLE after the laser hair removal.

## STATISTICAL ANALYSIS

Collected data was inserted into SPSS version 25. We reported quantitative variables (age, disease duration, and SLEDAI-2K score) by mean and standard deviation, and we used paired T-test to compare these variables before and after treatment. Qualitative variables (sex, marital status, education level, Skin phenotype, etc) were expressed as numbers and percent and compared by Chi-square test. A P-value less than 0.05 was considered to be statistically significant.

## RESULTS

20 patients with SLE were enrolled in the study. Most of the participants were female (90%). The mean age of the participants was 32.65 and the standard deviation (SD) of 5.38. The mean disease duration was 7.40 years with an SD of 4.48 (the shortest one year, and the longest 16 years). The detailed data of the demographical and clinical variables are shown in **Table 1**. The most common skin phenotypes based on the Fitzpatrick classification were II and III types. The mean SLEDAI-2K score was 6.95 with an SD of 8.64 before treatment.

**Table 1.** Demographic and clinical characteristics.

**Variables**	**Number (percent)**	**Mean (standard deviation)**

Gender		
Female	18 (90)	
Male	2 (10)	

Age		32.65 (5.38)

Education level		
Uneducated	0	
Less than or equal to high school	1 (5)	
High school graduate	11 (55)	
College or University	8 (40)	

Marital status		
Single	6 (30)	
Married	14 (20)	

Disease duration (years)		7.40 (4.48)

Underlying disease		
Hypothyroidism	5 (25)	
No underlying disease	15 (75)	

Medications		
Prednisolone (dose=< 7.5mg/d)	19 (95)	
Hydroxychloroquine	18 (90)	
Azathioprine	8 (40)	
Mycophenolate Mofetil	4 (20)	

Skin type		
I	1 (5)	
II	7 (35)	
III	7 (35)	
IV	5 (25)	
V	0	
VI	0	

Hair thickness		
Low	1 (5)	
Medium	13 (65)	
High	6 (30)	

SLEDAI-2K score		6.95 (8.64)

**Table 2** compares the SLEDAI-2K score. serum levels of Anti-ds-DNA, C3, C4, and CH50 complement levels, white blood cell, and platelet counts before and after six sessions of laser. The results were not statistically significant.

**Table 2.** Comparison of lupus activity before and after hair removal laser course.

**Clinical variables**	**Before laser therapy**	**After laser therapy**	**P-value**
SLEDAI-2K (number and percent of patients)			
No activity (score=0–3)	9 (45)	10 (50)	
Mild to Moderate (score=4–12)	11 (55)	10 (50)	
High activity (score>12)	0	0	>0.99

Anti-ds-DNA antibody level			
Normal	12 (60)	12 (60)	
Increased	8 (40)	8 (40)	>0.99

CH50 level			
Normal	18 (85)	18 (85)	
Increased	3 (15)	3 (15)	>0.99

C3 complement level (mean, SD)	101.52, 29.82	103.08, 38, 65	0.80

C4 complement level (mean, SD)	18.53, 10.79	20.03, 12.96	0.83

White blood cell count	5.72, 1.82	6.90, 2.53	0.21

Platelet count	245.22, 46.33	286.0, 30.19	0.50

Our patients reported no serious adverse events after the last sessions. Mild erythema and pain after each session were the only complications that resolved a few hours after laser therapy, and there were no SLE exacerbations or new flares during the study.

## DISCUSSION

Laser is an effective and safe method for the permanent reduction of unwanted hair. All available lasers including intense pulsed light (IPL), ruby, alexandrite, diode, Nd:YAG emit red or near the infra-red spectrum of light. Each laser is suitable for a skin type. For example, alexandrite and ruby lasers are more suitable for Fitzpatrick skin types I to III and diode for types III to V. The mechanism of action of laser hair removal is that the red and infrared light is absorbed by the melanin in the hair follicles and converted to heat energy which destroys hair follicles and stem cells located at the hair bulge. Since melanin is found in basal epidermal cells ultrastructural damage has been detected in these cells in the absence of obvious macroscopic injury. Individuals with darker skin have a higher rate of thermally induced adverse effects due to the higher rate of light absorption by the epidermal melanin. In addition to skin phonotype, other parameters such as wavelength, pulse duration, and energy density should be selected correctly to target the hair follicles without harming the adjacent tissue.^4,7–9^ The known adverse effects include erythema, oedema, pain, heat burns, blisters, scarring, hyperpigmentation, hypopigmentation, and paradoxical hypertrichosis. As we mentioned, patients with darker skin are at higher risk of thermal injury due to the higher amount of epidermal melanin. Cooling devices protect the epidermis from damage in these patients.^10^

Alexandrite laser, which was used in our study, emits a 755 nm wavelength that is in the range of infra-red light. It is more suitable for Fitzpatrick skin types II to III. Longer wavelengths and pulse durations, along with using cooling devices are helpful to reduce adverse effects in darker skin phenotype.^11^ Since 70% of our patients had Fitzpatrick skin types II and III, the alexandrite laser was an appropriate choice.

Regarding the effect of hair removal laser on the production of reactive oxygen species, Sorg et al. and Zastrow et al.^12,13^ showed that IPL induces elevated levels of lipid peroxide which develops from oxidative degradation of lipids in cellular membranes. It is well-established that ultraviolet (UV) wavelength which is below 400 nm leads to DNA alteration. All the available laser devices produce wavelengths above 500 nm (non-ion-ising radiation) which is not harmful to DNA strands. Sorg et al. investigated the effect of IPL irradiation on the Thymine Dimers molecular lesions formation from thymine or cytosine bases in DNA—showing the photon-induced damage. They exposed nine individuals with IPL light wavelength range of 520–750 nm, 9 J/cm^2^ fluence, and pulse duration of 2.5 msec, and histological investigations showed no evidence of Thymine Dimer production when compared to control or UV-A-exposed sites.

Systemic lupus erythematosus is a multi-systemic chronic inflammatory disease. While the exact aetiology of the disease is unknown, it is assumed that some environmental factors might cause SLE or trigger disease flare-ups in a genetically predisposed individual. Ultraviolet light is a well-known environmental trigger for SLE exacerbations, and it could also trigger disease onset. UV light induces apoptosis of dermal cells such as keratinocytes leading to the release of a large amount of autoantigens and pro-inflammatory cytokines. In addition, it may alter DNA and intracellular proteins to make them antigenic. The ability to promote autoimmunity is dose-dependent. High doses of UV induce DNA fragmentation and necrosis of epidermal cells.^1–3^

As we mentioned above, despite the selectivity for melanin in hair follicles, a hair removal laser might cause damage to the surrounding skin. Damage to the skin cells and release of autoantigens is a potent trigger for SLE which is also caused by UV light. Therefore, there is a concern about the possibility of an increase in SLE activity by hair removal laser. We conducted the present survey and enrolled 20 patients with SLE to investigate the effect of six monthly sessions of Alexandrite laser hair removal on the activity of SLE. SLEDAI-2K is a valid tool to identify clinically significant changes in SLE disease activity.^14,15^ We compared the mean SLEDA score before and after the laser hair removal course and observed differences were not statistically significant (SLEDAI-2K score: 6.95 vs 5.70. P=0.19). The changes in the serum levels of Anti-ds-DNA, C3, and C4 complement, platelet, and WBC counts were not significant. Six monthly sessions of laser hair removal were safe in our study group.

The main limitation of our study was the small sample size. Therefore, further studies with larger sample sizes and other types of lasers are required to determine the exact effect of hair removal lasers on the SLE activity. The main strength of the study was that as far as we know we conducted the first study to investigate the effect of hair removal laser on SLE activity. In addition, we used SLEDAI-2K which is a valid tool to evaluate disease activity.

In conclusion, six monthly sessions of laser hair removal with Alexandrite laser had no significant effect on SLE activity measured by SLEDAI-2K. Laser hair removal could be safe in patients with SLE if it is compatible with the skin phenotype and correct laser parameters are used.

## CONFLICT OF INTEREST

The authors declare no conflict of interest.

## AUTHOR CONTRIBUTIONS

SA and ES conceived the idea and designed the study. ES was responsible for data collection and performing laser procedures. MR was responsible for data analysis. SA and DM drafted the article. All the authors read and approved the final manuscript.

## FUNDING

This research did not receive any specific funding.

## ETHICS APPROVAL AND CONSENT TO PARTICIPATE

The Local Clinical Research Ethics Committee approved the study protocol. Informed written consent was obtained from the participants.

## AVAILABILITY OF DATA AND MATERIALS

Available if requested.
